# Graded/Gradient Porous Biomaterials

**DOI:** 10.3390/ma3010026

**Published:** 2009-12-25

**Authors:** Xigeng Miao, Dan Sun

**Affiliations:** 1 Institute of Health and Biomedical Innovation, Queensland University of Technology, 60 Musk Avenue, Kelvin Grove, QLD 4059, Australia; 2 NIBEC, University of Ulster, Jordanstown Campus, Co. Antrim, NI, BT37 0QB, Northern Ireland, UK; E-Mail: D.Sun@ulster.ac.uk (D.S.)

**Keywords:** graded, gradient, porosity, pore size, bone, biomaterials

## Abstract

Biomaterials include bioceramics, biometals, biopolymers and biocomposites and they play important roles in the replacement and regeneration of human tissues. However, dense bioceramics and dense biometals pose the problem of stress shielding due to their high Young’s moduli compared to those of bones. On the other hand, porous biomaterials exhibit the potential of bone ingrowth, which will depend on porous parameters such as pore size, pore interconnectivity, and porosity. Unfortunately, a highly porous biomaterial results in poor mechanical properties. To optimise the mechanical and the biological properties, porous biomaterials with graded/gradient porosity, pores size, and/or composition have been developed. Graded/gradient porous biomaterials have many advantages over graded/gradient dense biomaterials and uniform or homogenous porous biomaterials. The internal pore surfaces of graded/gradient porous biomaterials can be modified with organic, inorganic, or biological coatings and the internal pores themselves can also be filled with biocompatible and biodegradable materials or living cells. However, graded/gradient porous biomaterials are generally more difficult to fabricate than uniform or homogenous porous biomaterials. With the development of cost-effective processing techniques, graded/gradient porous biomaterials can find wide applications in bone defect filling, implant fixation, bone replacement, drug delivery, and tissue engineering.

## 1. Introduction

As a special group of dense composite materials, functionally graded/gradient materials (FGMs) have about two decades of history. FGMs are materials with purposely designed continuous change (gradient) or step-wise change (graded) in microstructure and properties. FGMs offer the advantage of tailoring materials with specific structural, compositional, morphological, and mechanical properties. The early research and development of FGMs was driven by the need of reducing the thermal stresses developed in thermal barrier coatings on high temperature alloys [1]. Now FGMs can be found in various material/phase combinations. FGMs have also been used as biomaterials for dental and orthopedic applications [2,3,4]. FGMs have been reviewed by some researchers [5,6].

Most engineering materials are in the dense form. However, porous materials are also very useful. Furnace lining bricks, filters, catalyst supports are some of the conventional porous engineering materials. As early as in 1989, Berdichevskii [7] reviewed the uses of porous ceramics. In 1996, Nettleship [8] reviewed some new applications of porous ceramics, namely, porous electrodes for solid oxide fuel cells, and membranes used for isolating bacteria in bioreactors. The fabrication methods for porous ceramics have been reviewed by several researchers [9,10,11,12,13,14].

The introduction of porosity into a biomaterial broadens the scope of applications in the biomedical field. Ceramic and polymeric scaffolds are examples of porous biomaterials. Ravaglioli *et al.* [15] reviewed the production, potential applications, and limitations of porous bioceramics. Simske *et al.* [16] reviewed the principal types of porous biomaterials used in bone replacement. Liu and Dixit published a book entitled "Porous Materials for Tissue Engineering" [17]. Yang *et al.* [18,19] reviewed porous polymers as the scaffolds for tissue engineering.

The pores in a porous biomaterial can have a variable pore size and a porosity distribution and thus porous biomaterials can take the graded/gradient porous form. In Simske *et al.*'s review [16], graded or gradient porous materials were mentioned and an influential statement was given: "We believe an area of future clinical research will be the construction of implants with gradients of porosity." Indeed, there has been a research focus in graded/gradient porous biomaterials. However, the focused topic has not been reviewed before, although Rice [20] reviewed graded porous engineering materials in 2002. It is the purpose of this review to deal with the porous biomaterials with graded/gradient porous structure and properties. This review attempts to summarise the current achievements in the area.

It should be noted that human body itself is highly graded/gradient in organisation. The architecture of bone is such that the resulting porosities are non-uniform in nature. This is readily apparent in the longitudinal cross-section of a long bone where the bone at the ends has the appearance of a sponge (cancellous or trabecular bone) while the bone in the middle of the bone shaft is rather dense or with a low porosity (cortical bone). Nonuniform porosity is apparent in bone even at the microscopic level. At this level, vascular channels (Haversian and Volksmann canals) are approximately 100–250 microns in diameter. Bone producing cells (in lacunae, 5–10 microns in diameter) and interconnecting fenestrations (canaliculi, 1–5 microns in diameter) are examples of the low end of the porosities present in bone.

## 2. Porous Structural Characteristics

**Figure 1:**
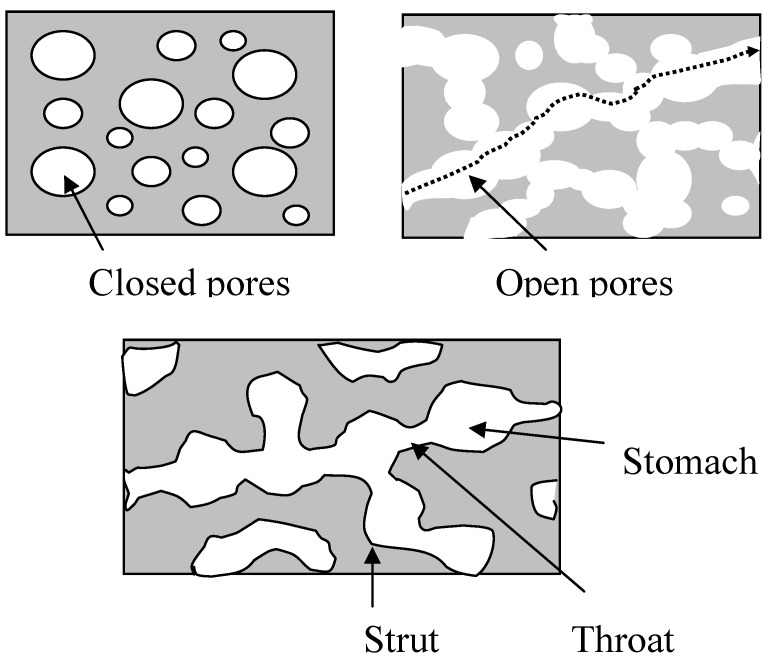
Schematic illustration showing the porous features of a porous biomaterial.

For all porous biomaterials, total porosity (= total pore volume/overall (or bulk) volume) is one of the important structural parameters. Total porosity has negative effect on the mechanical properties. However, total porosity alone does not have a direct relationship with cell/tissue ingrowth, as pore size and pore interconnectivity are more important for cell/tissue ingrowth. A porous biomaterial may have both closed (isolated) pores and open (connected) pores. Connected pores look like tunnels and are accessible by gas, liquid, and particulate suspensions. Pore interconnectivity is defined as the fraction of open pore volume/(open pore volume + closed pore volume) = open porosity/total porosity, where open porosity = open pore volume/bulk volume. Apart from the total porosity and the pore interconnectivity, pore size and pore size distribution are also important. Depending on pore size, micropores (e.g., <10 microns) and macropores (>50 microns) are used in describing the porous biomaterials. Micropores are often present in the struts of the porous biomaterials and the connected macropores are often inhomogeneous along the pathway. Thus, there is a throat size and stomach size (Figure 1). If the throat size is too small, cells and/or tissues may not be able to migrate or grow into the pores. Mechanical properties and cell/tissue ingrowth behaviour depend on the pore size, porosity, and pore interconnectivity in different ways. Unfortunately, some processing methods for porous biomateruials do not grantee high pore interconnectivity and large enough throat size for cell/tissue ingrowth, although total porosity can be achieved easily.

## 3. Types and Potential Applications of Graded/Gradient Porous Biomaterials

Most porous biomaterials developed and studied so far are homogeneous in terms of pore size distribution, porosity distribution, composition, and mechanical properties. However, there are needs and interests to produce heterogeneous porous biomaterials. Heterogeneity in pore size, porosity, composition, and mechanical property can result in optimized structural, biological, and mechanical functionalities of porous biomaterials. Graded/gradient porous biomaterials belong to heterogeneous porous biomaterials and are defined as those porous biomaterials with graded/gradient pore size/porosity throughout the porous structures. Another case is the compositionally graded or gradient porous biomaterials, where the pore size, the porosity and the composition can all be graded or gradient. Some examples of biomaterials with graded/gradient pore size, porosity, composition, and thus mechanical properties are shown in Table 1.

**Table 1:** Examples of graded/gradient porous biomaterials and their potential applications.

Biomaterials	Potential applications	References
Graded porous Ti	Permanent skeletal replacement implants with minimized stress shielding	[21]
Gradient porous Ti	Dental implants with osteointegration	[22]
Graded PEGT/PBT *	Tissue engineered cartilage with zonal organization	[23]
Gradient porous PCL *	Fundamental study of the effect of pore size on cell or tissue ingrowth	[24]
Graded porous HA *	Mimicking bimodal structure of bone (cortical and cancellous)	[25]
Graded porous HA	For mechanical strength and cell/tissue ingrowth	[26]
Graded porous HA	Tissue engineering scaffolds and drug delivery systems	[27]
Graded porous HA	Cages for spinal fusion requiring mechanical strength and osteointegration	[28]
Graded porous Al_2_O_3_-ZrO_2_ composite	For both mechanical strength and osteoconductivity	[29]
Compositionally graded porous (d,l-PLGA + l-PLA) on (l-PLGA + TCP) *	Articular cartilage repair and integration with subchondral bone	[30]
Compositionally graded porous collagen on PLGA-collagen	Osteochondral (*i.e.,* bone-cartilage) tissue engineering	[31]

* PEGT/PBT: poly(ethylene glycol)-terephthalate-poly-(butyleneterephthalate). PCL: poly(ɛ-caprolactone). HA: hydroxyapatite. PLGA: poly(lactic-co-glycolic acid). PLA: polylactide. TCP: tricalcium phosphate.

**Figure 2:**
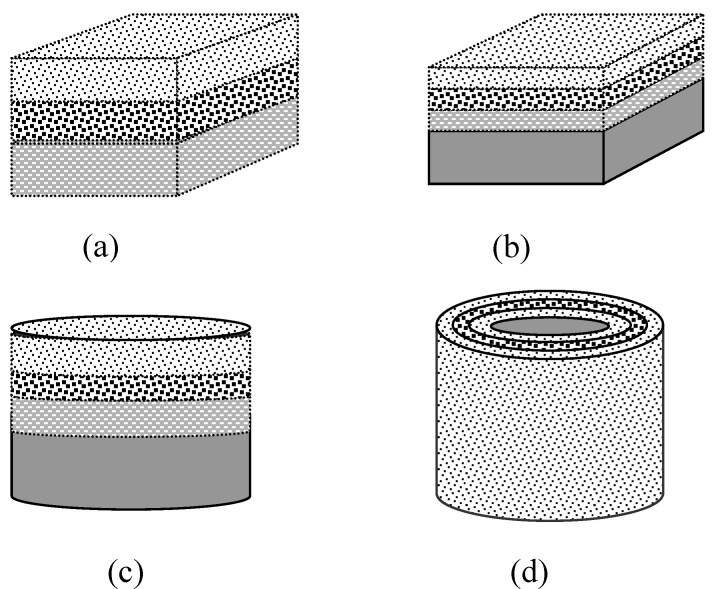
Schematic diagrams showing the basic configurations and shapes of the graded/gradient porous biomaterials: (a) graded porous block; (b) graded porous block with a dense part; (c) graded porous disk with a dense part; (d) graded porous layer on a cylindrical dense core.

Graded/gradient porous biomaterials can also be grouped according to the overall shape and the structural configuration. The basic shapes are rectangular blocks and cylinders (or disks). For the cylindrical shape, there are configurations of dense core-porous layer, less porous core-more porous layer, dense layer-porous core, and less porous layer-more porous core. For the rectangular shape, in the gradient direction *i.e.,* the direction with varying porosity, pore size, or composition, there are configurations of porous top-dense bottom (same as porous bottom-dense top), porous top-dense center-porous bottom, dense top-porous center-dense bottom, *etc.* These basic shapes and configurations can be made into more shapes if the porous biomaterials can be machined or trimmed. Different applications require different configurations and shapes. For example, dense core-porous layer is suitable for implants of a high mechanical strength and with bone ingrowth for stabilisation, whereas less porous layer-more porous core structure can be used for drug delivery systems. Furthermore, the porous top-dense bottom can be shaped into implants of articulate surfaces for wear resistance and with porous ends for bone ingrowth fixation. Finally, the dense top-porous center-dense bottom will mimic the structure of head skull. Some examples of the shapes and configurations are shown in Figure 2.

**Figure 3:**
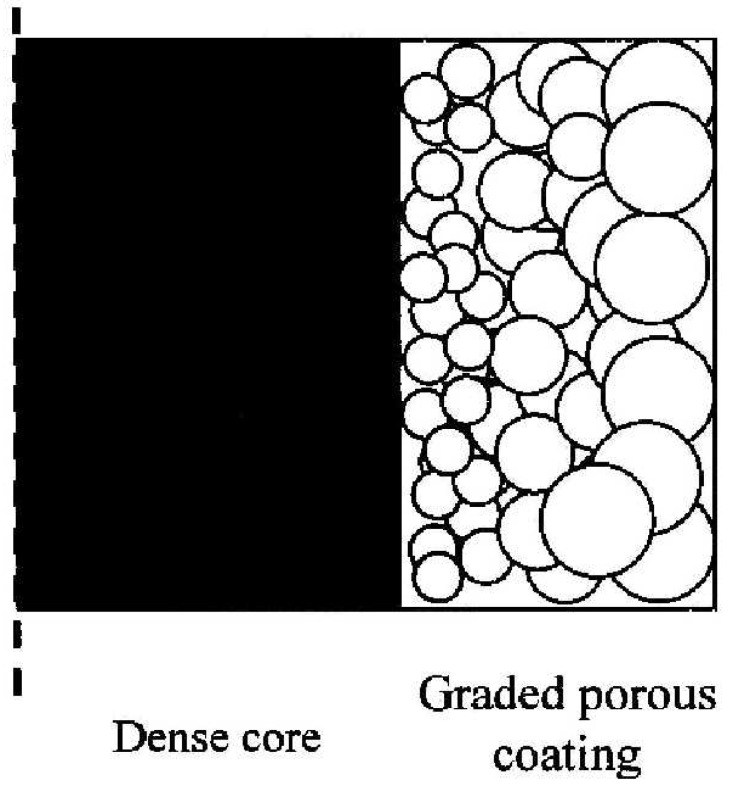
Schematic illustration showing the graded porous layer on the dense core.

**Figure 4:**
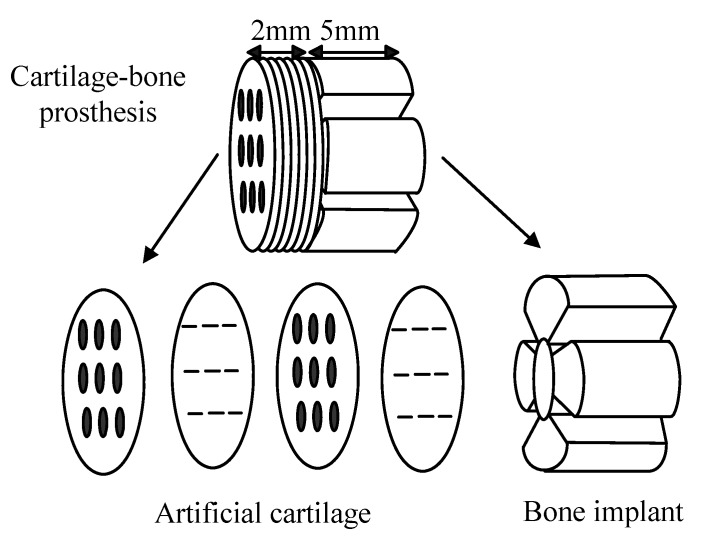
Schematic illustration showing a graded porous implant.

Some examples of the biomedical applications of graded/gradient porous biomaterials are cited below:

(1) According to Collier [32], the femoral cap for the hip joint could be made of Co-Cr alloy with functionally graded porosities and pore sizes. The graded pore sizes were from 500 μm to 100 μm in the cap part facing the acetabular bone. The other part facing the articulating surface could have graded pores of pore sizes < 50 μm. The small pores would prevent bone ingrowth but permit cartilage ingrowth whereas large pores were for bone ingrowth after implantation for biological fixation. Similarly, functionally graded porous materials could replace damaged portion in the femoral head. Although the above implants were made of metals, the implants can be also made of graded porous ceramics, such as alumina.

(2) Solid metals like Ti alloy or Co-Cr alloy are traditionally used as hip stems. The problem is the stress shielding caused by the mismatch in stiffness between the implants and the bone. One way to minimise the stress shielding is to prepare a functionally graded porous coating on a conventional solid stem (Figure 3). In the graded porous coating, the graded porosity increases from the solid stem outward, resulting in stiffness decreasing down to the level of the cortical bone. In this way, stress shielding can be minimised [33].

(3) Graded porous implants (Figure 4) can be used for repairing bone-cartilage complex tissue. The large pore sized part is to be implanted into bone for bone ingrowth, whereas the small pore sized part is to allow cartilage to grow in. In other words, the graded porous implant can be used to select or promote attachment of specific cell types on and in the implant prior to and/or after implantation. The part for bone ingrowth and the part for cartilage ingrowth can be made of different materials. The gradient of materials properties may be from that which is suitable for load bearing to one which is suitable for soft tissue regeneration [34].

## 4. Characteristics and Advantages of Graded/Gradient Porous Biomaterials

Smiske *et al.* [14] mentioned four levels of pore sizes, with each level having its own functions. The first level (1–100 microns) is typically observed in porous bone structure. Biomimetic principle dictates that this level of pore sizes in biomaterials is necessary. The second level (100–350 microns) is optimum for bony ingrowth. While larger pore sizes (350–1,000 microns) tend to decrease the mechanical strength, they are useful to lower the Young’s modulus of a porous implant to reduce the stress shielding. Finally, for mechanical attachment of a porous implant by a wiring-like suture during the medical surgery, large pores (350–3,500 microns) would be useful. From the above statements, one can see that uniform porous biomaterials with either of the four levels of porosities would have limitations in functions. If a porous implant is to have all the functions, then the implant should not be a uniform one or a dense one, but rather it should be one with a graded/gradient porosity. Ideally, the implant with a graded/gradient porosity should be such that the largest porosity is near the implant surface facing the host bone and the porosity continually scales down until it reaches either a solid core material for loading bearing applications or a porous core for no or low loading applications.

Dense biomaterials have high mechanical properties but poor bone ingrowth properties. On the contrary, porous materials provide good biological performance but have a low mechanical strength. Porous materials have a large ratio of pore surface area to bulk volume, thus there is a large surface area for cells to attach and grow on. It is known that most bone forming cells grow on a substratum surface rather than grow in a suspended manner in the cell culture medium. The large pore surface area also means that there is a large bone-material interfacial bonding area if the porous material involved is bioactive. Furthermore, bone growth into a porous implant leads to the stabilization of the implant (*i.e.,* biological fixation). Interconnected porosity also acts like an organisation of vascular canals that can ensure the supply of blood and nutrients for the viability of bone. Finally, porous biomaterials have much reduced Young's moduli compared to the dense counterparts, thus it is possible to match the Young's moduli of porous implants with those of bones. This match in Young’s modulus serves to minimise the problem of stress shielding. The graded/gradient porous biomaterials that are stressed in this review will combine the advantages and minimise the limitations of both the dense and porous biomaterials.

Graded/gradient porous biomaterials have other advantages. For example, an implant of the graded/gradient porosity will mimic the natural porous structure of bone more closely than the uniform porous biomaterials. It is known that the bone cross-section from cancellous to cortical bone is non-uniform in porosity and pore size. Thus, a damaged bone containing both cancellous and cortical bone can be better replaced by a graded/gradient porous implant based on the idea of biomimetic approach.

Implants can have varying porosities in different regions of the implants for optimizing ingrowth of different tissues and cells. It is known that different sizes of pores are suitable for ingrowth of different tissues. For example, 5–15 μm for fibroblast ingrowth, 70–120 μm for chondrocyte ingrowth, 100–400 μm for bone regeneration, depending on the porosity as well as scaffold materials [24]. The uniform porous materials only allow one particular tissue type to grow in, depending on pore size, whereas graded/gradient porous materials can repair and reconstruct two or more different tissues simultaneously, as the different regions provide different microenvironments. In addition, graded/gradient porous biomaterials can be used as a powerful tool to study the interactions between cells and porosity and/or pores size, as no separate and individual uniform scaffolds are needed, and the effect of pore size can be effectively examined using one graded scaffold [24], resulting in experimental convenience and cost saving.

Graded/gradient porous biomaterials can be used as scaffolds that can lead to a new tissue mimicking a natural tissue structure. For example, a cell density gradient was formed after seeding chondrocytes in the graded PEGT/PBT scaffold. After *in vitro* cell culture, the extracellular matrices such as collagen type II showed inhomogeneous distribution. The inhomogeneous distribution of the cells and the extracellular matrices are present in the natural articulating cartilage, which has the zonal cell, structural, and mechanical organization [23]. In addition, the morphology of the scaffolds can affect the vascularisation and the structure of a tissue.

Graded/gradient porous implants optimize the ability of the implants to withstand varying mechanical loads at specific regions of the implants and subsequently minimise the stress shielding problem. For example, a graded material can be used for acetabular cup prosthesis where the dense part has good mechanical properties and wear performance, whereas the back side of the prosthesis with a porosity gradient is for bone ingrowth and biological fixation, resulting in minimal stress shielding [32].

It is known that bone ingrowth into a porous biomaterial is not uniform both through the depth of the implant and over the time period after the implantation. Initially bone grows into the pore spaces very fast but the growth rate slows down with the maturation and remodelling of the bone. Thus graded/gradient porosity in the implant can match or cater for the growth rate of bone ingrowth into the porous implant. In fact, regions of high porosities should be allowed to face the host bone so that initial bone ingrowth and maturation occurs, whereas the parts of low porosities should be in the implant interiors where a bone needs longer time to develop.

## 5. Fabrication of Graded/Gradient Porous Biomaterials

The applications of porous materials depend on the pore size and other porous parameters and the fabrication of porous materials with different pore sizes requires different manufacturing processes. The processing methods suitable for uniform porous biomaterials may not always be suitable for graded/gradient porous biomaterials. Fortunately, several methods have been developed for the fabrication of graded/gradient porous biomaterials, as summarised in Table 2.

**Table 2:** Fabrication methods and their characteristics.

Fabrication methods	Porous characteristics and other comments	References
Sintering of graded particles	Good pore interconnectivity, but small pore neck size and low porosity	[21]
Pulsed electric current sintering (PECS)	Good pore interconnectivity, but low porosity and expansive	[22]
Vacuum infiltration	Good pore interconnectivity and good pore size control, but has dimensional limitation	[35]
Pressure filtration of mixed particles	Controllable porosity, but poor pore interconnectivity and complicated process	[36,37]
Infiltration of compression-moulded sponge	High pore interconnectivity, graded pore size, porosity, and pore shape.	[38,39]
Multiple and differentiated impregnation	Good pore interconnectivity, graded porosity and pore size, but not good for small samples	[25]
Multiple tape casting and lamination	Controllable pore size and porosity, but poor pore interconnectivity especially on the interfaces	[26]
Solid free form fabrication	Good pore interconnectivity, controllable porosity, but difficult to achieve small pore sizes	[23,30]
Centrifugation of a suspension and freeze drying	Gradient porosity and pore size, but suitable for limited material systems	[24]
Introduction of a graded biodegradable phase	Absence of macropores before implantation and able to support mechanical load	[40,41]
Electrospraying into a template	Good pore interconnectivity, but difficult to control the structural regularity	[45]
Freeze-casting	Good pore interconnectivity but difficult to control the temperature gradient	[46]
Combined method	Resulting in more complex structures	[47]
Microspheres for scaffolds	Similar to the method of sintering of graded particles, and various methods developed for polymer microspheres.	[48,49,50,51,52,53,54,55,56,57,58,59,60,61]

### 5.1. Sintering of Graded Particles

**Figure 5:**
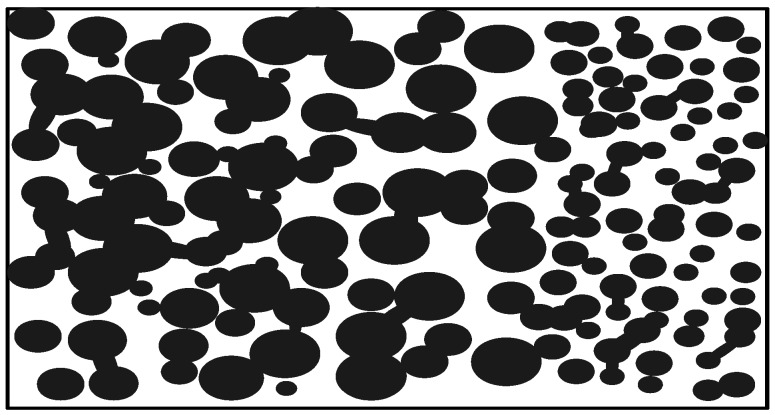
Schematic illustration showing the graded porous structure prepared by the powder metallurgy method.

This method relies on the partial sintering of ceramic or metal or polymer powder. The powder is normally in the form of granules with different particle sizes. The granules are packed into a green shape (*i.e.,* the compact of particles before sintering), forming a gradient in particle size. The green shape thus packed also simultaneously contains a gradient in porosity. To impart mechanical strength, the contacting areas among the granules must be strongly joined through sintering at a high temperature. For example, by using Ti particles of different particle sizes and by adding silicon for liquid-phase sintering, functionally graded porous Ti material with improved neck geometry was prepared (Figure 5) [21]. The Young's modulus varied from 5 to 80 GPa along the gradient direction, which covered that of cortical bone (10–29 GPa). Due to the gradient in porosity, the Young's modulus of the material was adapted to the elastic properties of bone so that the stress shielding could be prevented. The material was designed for a permanent skeletal replacement implant with improved structural compatibility. Obviously, this method relies on the availability of the staring powders of different particle sizes. Fortunately, there have been several methods to produce microspheres or spherical particles, as will be mentioned at the end of this section.

### 5.2. Pulsed Electric Current Sintering (PECS)

PECS (also called spark plasma sintering) is like a hot pressing process, as a graphite mould (or die) and a punch are used to press ceramic or metal particles in the die. The high temperature needed to sinter the particles is caused by the heat due to the direct current passing through the surfaces of the contacting particles. The porous structure of the sintered body is thus dependent on the applied pressure, the applied power, and the sintering time. Due to the fast heating, PECS allows the fast formation of strong necks among the particles and thus a highly porous sintered structure can be formed without much volume shrinkage. If a temperature gradient can be established, then a gradient porous structure can be formed due to the varying sintering conditions. Using PECS, Suk *et al.* [22] produced a porous material with a porosity gradient by creating a temperature gradient in the packed particles and the temperature gradient was generated by varying the wall thickness of the die. The linearly increased wall thickness led to a temperature gradient to be established along the longitudinal direction inside the die.

PECS method requires expensive equipment, and the resultant samples are limited to small dimensions and simple shapes. These are the disadvantages of PECS compared with the above mentioned powder metallurgy method. However, power metallurgy method tends to lead to a deformed or distorted shape after pressureless sintering due to the particle size gradient causing different sintering shrinkage. In PECS the porosity gradient is formed due to the temperature gradient, whereas in the powder metallurgy method, the porosity gradient is caused by the gradient particle size. Obviously, pressureless sintering of green compacts of spatially stacked and graded particles (either ceramic or metallic) as the powder metallurgy method is more practical for the formation of a porous structure of a graded/gradient pore size or porosity.

### 5.3. Vacuum Infiltration

**Figure 6:**
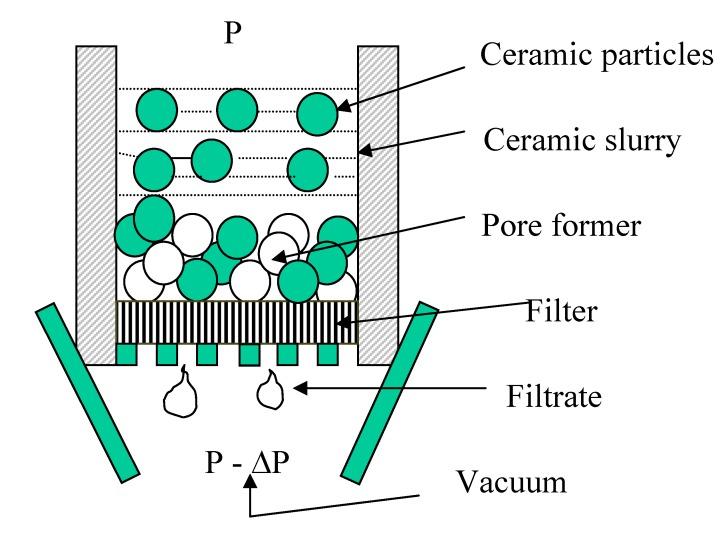
Schematic illustration showing the formation of a green compact among the pore space within the polymer bead bed (P: pressure drop; ∆P: pressure drop).

This method is based on the infiltration of a ceramic slurry into a porous polymer body using a vacuum system (Figure 6). In order to obtain a final porous ceramic structure with a pore size gradient, the porous polymer body is made to have graded pore sizes, which can be formed by packing loose polymer beads of different particle sizes in a sequential manner. The ceramic slurry is formed based on the colloidal processing approach; the ceramic particles are dispersed in an aqueous solution with a surfactant so that the flowability of the slip (or slurry) is achieved. During the infiltration of the slip, the ceramic particles are packed inside the pores among the polymer beads. After drying, the ceramic particle—polymer bead mixture is fired to remove the polymer beads and densify the remaining ceramic struts. Consequently, a graded porous ceramic body can be prepared, which is a negative replica of the initial graded porous polymer template. Miao *et al.* [35] used expanded polystyrene (EPS) beads with different sizes to form graded porous polymer templates and prepare graded porous yttria-stabilized zirconia ceramics. The vacuum infiltration method can be used to prepare other bioceramics such as hydroxyapatite, bioactive glass, *etc.* The advantage of using the expanded polystyrene rather than dense polystyrene beads lies in the less mass and less or no expansion during the stage of removing the beads by burning. Otherwise, cracks or shape distortion will be observed in the formed porous ceramic body. While the graded pore size is easy to achieve with this method, there is a need to press the EPS beads to keep the template shape and also allow enough contacting area between beads, which will lead to good pore interconnectivity. Vacuum infiltration can also be used to produce graded porous polymer scaffolds. For example, a polymer solution (a polymer dissolved in an organic solvent) can penetrate into a graded template of salt particles or wax particles, followed by evaporation and template removal.

### 5.4. Pressure Filtration of Mixed Particles

**Figure 7:**
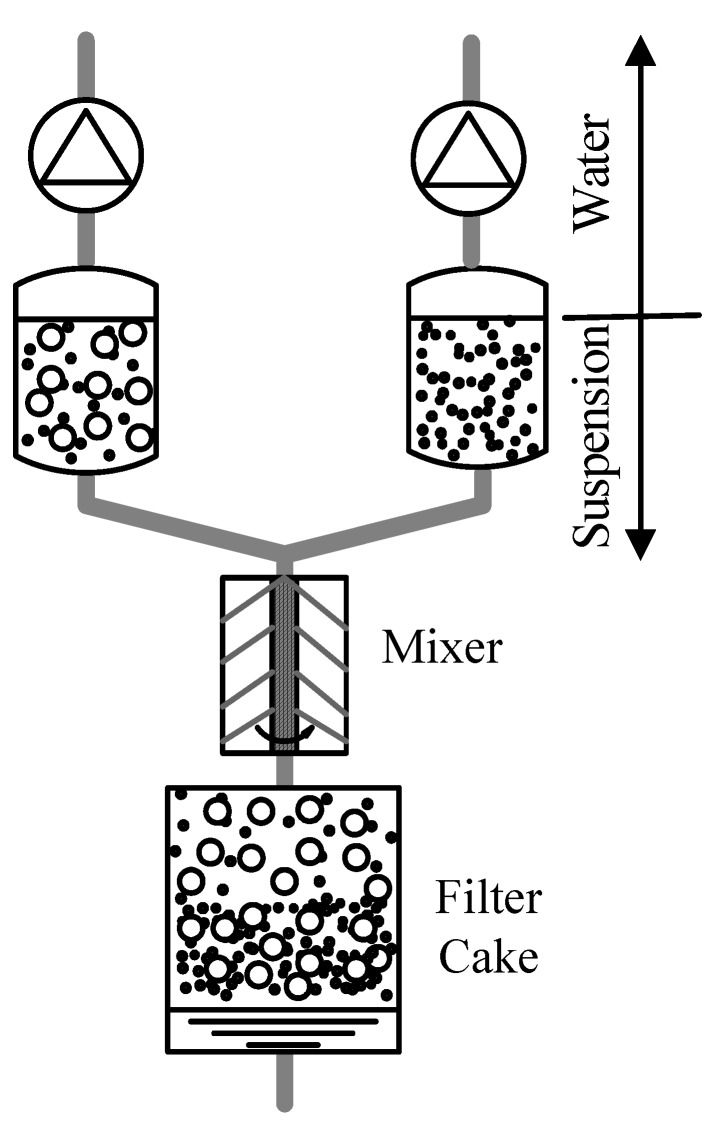
Schematic diagram showing the formation of a graded filter cake.

Graded porous hydroxyapatite ceramics were prepared by mixing a hydroxyapatite suspension and a carbonaceous particle suspension during the filtration process [36]. The composition profile of the filter cake could be controlled in the direction of cake thickness (Figure 7). After drying and consolidation, the cake was sintered in an oxidising atmosphere, resulting in graded porous sintered hydroxyapatite. This method differs from the above vacuum infiltration method in that there is no gradient in the carbonaceous particle (pore former) size and there is a compositional gradient of the carbonaceous particles, leading to a porosity gradient rather than a pore size gradient. There is also a limitation that the carbonaceous particles may not be in close contact, leading poor connectivity of the pores. It should be noted that the device shown in Figure 7 is only suitable for flat disc shapes. To produce porous tubes with a porosity gradient both in the radial direction and in the axial direction, another pressure filtration apparatus was used, where a ceramic slurry and a ceramic-pore former slurry were mixed at controlled and variable ratios [37]. Specifically, the mixed slurries were compacted via filtration assisted by gas pressure. The axial porosity gradient was controlled by the mixing ratios that were changing gradually during the process. On the other hand, the radial porosity gradient was controlled by the differential pressure drop created by the segmental filters, flow valves and the rotating cylinders.

### 5.5. Infiltration of Compression-Moulded Sponge

**Figure 8:**
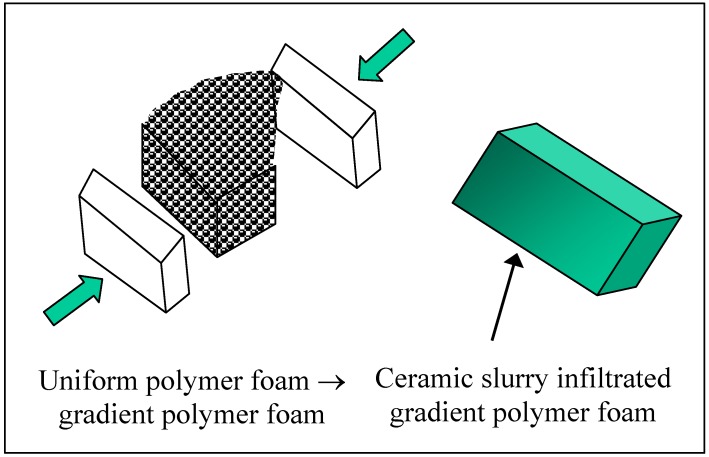
Schematic illustration showing the compression-moulding process for obtaining a gradient sponge preform.

A new method was used to produce gradient porous ceramics by using the deformed polyurethane foams with a porosity gradient (Figure 8) [38,39]. Specifically, reticulated polyurethane sponges having a pore size of ~31pores/cm and a porosity of 98.5% were sectioned to various thicknesses and shapes. The sectioned sponge foams were subsequently placed in aluminium moulds and compression moulded at 110 °C for 6 hours. This step of compression moulding effectively deformed the polymer sponge as well as fixed the open pore volume of the sponge. Compression moulding a wedge-shaped sponge into a planar geometry led to a sponge of a continuous porosity gradient produced. On the other hand, a stable suspension of Al_2_O_3_ powder was made with the aid of an organic dispersant. Then the preformed sponge was inserted into a pneumatic pressure infiltration device, and the Al_2_O_3_ slurry was pressure cast into the pore spaces between sponge networks. The castings were dried at room temperature in air for 5 days prior to heat treatment. A two-step heat treatment was then used to first pyrolyse the sponge and subsequently sinter the body of packed ceramic particles (or green body), resulting in gradient porous alumina ceramics with relatively small pore sizes but a high pore interconnectivity.

The slurry (or colloidal) infiltration of a preformed sponge is for porous ceramics with a low porosity because the pores of the sponge are filled with ceramic particles. However, if the pores are partially filled or if the struts of the sponge are coated with ceramic particles, then gradient/graded porous ceramics with a high porosity can be obtained. For example, dip coating of gradient polyurethane foams with a 45S5 Bioglass slurry was used to produce gradient 45S5 Bioglass^®^-derived glass–ceramic scaffolds for potential application in bone tissue engineering [43]. Specifically speaking, different types of template scaffolds with a continuous or stepwise gradient of porosity were produced using polyurethane (PU) foams that were preformed at 200 °C for 30 mins. Then they were dipped (as in a dip coating process) rather than fully infiltrated in a 45S5 Bioglass^®^-based slurry and subsequently heat treated in a chamber furnace up to 1,100 °C. During heating, the organic phase was burned out and the glass compact was densified and accompanied by crystallisation, leading to highly porous glass-ceramic scaffolds with different shapes and porosity profiles. The obtained scaffolds had highly interconnected porous structures with specific porosity gradients.

### 5.6. Multiple and Differentiated Impregnation

Graded porous hydroxyapatite (HA) was prepared by a multiple and differentiated impregnation method [25]. In brief, a cellulose sponge was first completely immersed in an HA slurry. After the immersion the sponge was coated with a layer of HA ceramic particles. Then the lower portion of the previously coated sponge was further immersed into the HA slurry so that the double coated portion had larger strut thickness than the single layer coated upper portion. Such immersion in the HA slurry could be repeated a few more times but at controlled locations to create a graded porous HA green body. After drying, the sponge was removed by firing up to 1,250 °C at a low heating rate. Porous HA ceramics with a graded porosity were thus prepared with the HA struts being densified. This method is similar to the slurry dipping of the graded/gradient sponges as mentioned in the above section, since the dip coating is used to generate a high porosity. However, the graded porosity here is made by the repeated but spatially different dip coating.

### 5.7. Multiple Tape-Casting and Lamination

**Figure 9:**
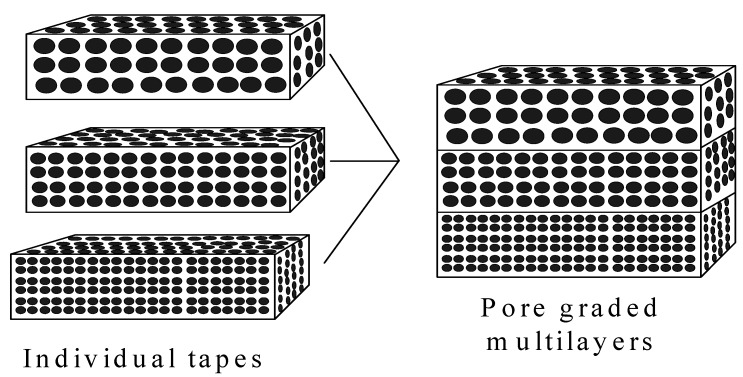
Schematic illustration of the fabrication of pore graded multilayers by lamination of individual tapes, manufactured by tape casting.

Werner *et al.* [26] produced graded porous hydroxyapatite materials by means of tape-casting and lamination (Figure 9). In this method, a hydroxyapatite slurry was mixed with a pore former. The mixed slurry was then cast into a tape. Using the same method, different tapes with different pore former sizes were prepared individually. The different tape layers were then laminated together. Firing was then done to remove the pore formers and sinter the hydroxyapatite particle compacts, resulting in graded porous hydroxyapatite. This method was also used to prepare graded porous HA with a dense part (core or layer) in order to improve the mechanical strength, as dense ceramics are much stronger than porous ceramics. However, as in the pressure infiltration of mixed particles, this multiple tape-casting also has the problem of poor connectivity of pores, although the pore size and the porosity are relatively easy to control. The lamination step also introduces additional discontinuity of the porosity on the interfaces between the stacked layers.

### 5.8. Solid Free Form Fabrication

Solid free form fabrication refers to a group of rather new technologies. It includes at least the following: selective laser sintering (SLS), fused deposition modelling (FDM), laminated object manufacturing (LOM), and three-dimensional printing (3DP). All the technologies employ the same basic five-steps, namely, 1. creating a CAD (computer-assisted design) model of the design, 2. converting the CAD model into the stereolithography (STL) format, 3. slicing the STL file into thin cross-sectional layers, 4. fabricating the model layer by layer with computer control, 5. cleaning and finishing the model. Solid free form fabrication gains good control over pore size (if large enough), shape, orientation and distribution including hierarchical and functionally graded pore structures. Solid free form fabrication is convenient for producing graded polymer scaffolds, as polymers can be shaped at low temperatures. However, this method is generally limited by the difficulty in obtaining fine pore sizes (e.g., <500 μm). To make graded porous ceramic scaffolds, there is a need to produce a graded porous polymer template, which will then be infiltrated with a ceramic slurry, followed by drying, burning and sintering. For example, composite scaffolds (Figure 4 shows an example) having a gradient of one or more of the following: materials (bioactive, bioinert, or biodegradable), macropores, micropores, or mechanical properties were prepared by the solid free form fabrication, especially the three-dimensional printing [34].

As another example, to fabricate 316L stainless steel implants with a pore gradient structure, a selective laser melting (SLM) technique (similar to SLS) was exploited on 316L powder compacts [44]. The microstructural feature of the laser scanned tracks (regions of sintered 316L particles), their densification, and the tensile strength of the SLM-produced samples prepared via different laser scan speeds were investigated. As the porosity was strongly influenced by the laser scan speed (time of melting), the scan speed was changed in a gradient manner and applied in every SLM layer for the purpose of producing a gradient porosity. The produced porous materials exhibited a gradually increased porosity and a reduced molten pool size along the gradient direction of the scan speed variation.

### 5.9. Centrifugation of a Suspension and Freeze Drying

Harley *et al.* [42] fabricated tubular scaffolds with a radial pore size gradient by a spinning technique. In this process, a collagen-glycosaminoglycan (CG) suspension in a cylindrical container was spun using a drilling machine. Due to the centrifugal force, there was different sedimentation of the CG fibres, resulting in gradients of CG content and water solvent. The gradient distribution of water and CG components was maintained by freezing using liquid nitrogen. Freeze drying was then used to remove the water, resulting in gradient porous CG scaffolds. A similar technique was used by Oh *et al.* [24], who produced PCL polymer fibrils and suspended them in a water solution. After centrifugation, freezing, and freeze drying, gradient porous PCL scaffolds were prepared. In the two examples, the fibers were fine enough in dimension and were not heavy compared to the density of water, which means the redistribution of the fibers even under centrifugation was relatively slow. If metal or ceramic fibers (having a much higher density than that of water) are used to produce gradient metal or ceramic sacffolds, then a polymer solution with a high viscosity should be used to suspend the heavy fibers, as the high viscosity can control the redistribution of the fine fibers even under centrifugation.

### 5.10. Introduction of a Graded Biodegradable Phase

Depending on chemical composition and crystal structure, calcium phosphates exhibit different dissolution rates in acidic solutions as well as different biodegradation rates *in vivo*. When two different calcium phosphates are properly mixed and sintered together, a functionally graded material having the two phases of the different dissolution rates can be prepared. By preferential acid etching *in vitro* or by selective biodegradation *in vivo*, graded porous calcium phosphate ceramics can be obtained.

For example, Wong *et al.* [40] prepared a powder of AlF_3_·3H_2_O-modified hydroxyapatite (FA) and a powder of β-tricalcium phosphate (TCP). Both powders were classified into different particle sizes: 150–300 μm, 106–150 μm, 75–106 μm, and <75 μm. The larger FA powders were mixed with larger TCP powders and the smaller FA powders were mixed with smaller TCP powder. However, the weight percentage of the FA in the largest particle size mixture was 50%, increasing to 80% for the smallest particle powder mixtures. After stacking the four powder mixtures layer by layer in a steel die, the compact of the mixed particles was formed and sintered at 1,300 °C for 2 hours. After being immersed in citric acid solution for some time, the beta TCP phase was leached out, leaving graded porosity, that is, functionally graded porous FA was prepared. The advantage of this latent graded composite is that it is initially dense and mechanically strong, but it becomes porous and is accompanied by tissue ingrowth. If the tissue growth rate is higher than the biodegradation rate, then the mechanical integrity of the graded composite and scaffold will be ensured.

This approach of using the biodegradation nature has also been adopted by other researchers. In order to reconstruct the complex skull defects with individually prefabricated CAD/CAM implants, a composite consisting of a polyester phase and a calcium phosphate phase was prepared with a graded composition [41]. The implants made of the composite led to a spatially guided tissue ingrowth since the components of the composite had different biodegradation rates.

### 5.11. Electrospraying into a Template

Electrospraying method involves the application of an electric field and is typically used to produce polymer microspheres by pulling a polymer solution or a ceramic suspension into droplets. Here electrospraying is not for producing microspheres but for directing the spatial deposition of ceramic particles onto a porous polymer template such as a polyurethane sponge; the ceramic particles are differently deposited onto the struts of the porous template, leading to a gradient ceramic mass distribution among the polymer template. Burning off the template and sintering the ceramic particles will thus form a gradient porosity in the final porous ceramic structure. For example, a structure of zirconia having a graded porosity was produced by electrospraying into a template, using a zirconia (ZrO_2_) suspension [45]. The pores on the outer surface, the innermost surface and the lengthwise cross sections were examined as a function of the spraying time. By varying the processing parameters (e.g., the spraying time, the sintering temperature and the polymer template), the porosity and the pore size of the final gradient ceramic body could be controlled. Structures with interconnected pore networks of pore size greater than 100 μm as well as scattered pores smaller than 10 μm in size could be obtained. It is obvious that the electrospraying method can be used to produce other bioactive ceramics such as hydroxyapatite with a gradient porosity. The electrospraying method also shares some degree of similarity to the multiple and differential impregnation method mentioned above.

### 5.12. Freeze-Casting

Water as a freezing vehicle is normally used in freeze-casting. More recently, camphene (C_10_H_16_), which has a melting point of ~ 45 °C has been used successfully as a freezing vehicle. Camphene can be frozen and easily sublimed at room temperature, allowing more flexibility in the process than water. A room temperature camphene based freeze-casting method [46] was used to fabricate hydroxyapatite/tricalcium phosphate (HA/TCP) ceramic scaffolds with a porosity gradient. In the freeze-casting, it was the temperature gradient generated in the cast mass, that influenced the pore size distribution. The lowest temperature corresponded to the largest size of camphene crystals and thus the largest pore size after the removal of the camphene crystals. The results of the study indicated that it was possible to manufacture porous HA/TCP bioceramics, with compressive strengths comparable to those of cancellous bones, using the freeze-casting technique, which could be of significant clinical interest.

### 5.13. Combined Method

Hsu *et al.* [47] produced a long bone-like porous structure with a dense wall and a porous core. Briefly, a porous polyurethane (PU) cylinder (of a larger pore size) and a hollow PU cylinder (of a smaller pore size) were obtained by a hot cut method. Then the PU cylinder of large pores was coated with hydroxyapatite/tricalcium phosphate (HA/TCP) particles by a slurry dipping method similar to the slurry coating of the compression moulded-sponge mentioned above. On the other hand, the hollow PU cylinder with small pores was infiltrated or impregnated with a concentrated HA/TCP slurry for making a dense wall. Then the two types of HA/TCP-containing cylinders were combined together by pressfitting (*i.e.,* inserting the slightly oversized and HA/TCP-coated PU cylinder into the HA/TCP-impregnated hollow PU cylinder). The pressfittng and the wet state of the HA/TCP mass were skilfully matched so that an interfacial defect or weak interface could be avoided after sintering the dried green sample at 1,280 °C. The final result of the process was a porous implant material with a graded pore structure similar to the bimodal structure of cortical and cancellous bone. This example indicates that combination or integration of some basic techniques can lead to a new method and a new porous structure. As another example, the vacuum infiltration method mentioned above can also be used to produce a porous structure of a dense core and a porous layer. In particular, the porous layer can be formed through the use of the EPS beads packed into a shell-like template, whereas the dense core can be formed by using a polyurethane foam cylinder placed inside the shell-like template. After ceramic slurry infiltration, burning and sintering, the dense core-porous layer structure will be formed and can be used as dental root implants.

### 5.14. Microspheres for Scaffolds

Microspheres can be made of a metal, ceramic, polymer or composite. Microspheres can also be dense or porous and have different sizes. Thus, it is possible to assemble the different microspheres to form scaffolds of a graded pore size, or a graded composition, *etc.* Two key issues here are the fabrication of the microspheres and the binding or sintering of the microspheres. Focus will be placed on polymer microspheres, as they can be assembled easily by sintering at a low temperature or be bonded by a solvent vapour or a hydrogel (e.g., alginate or fibrin) [48,49]. The methods of fabrication of the polymer microspheres (porous) will be briefed below.

Synthetic polymer microspheres with porosity can be produced by a water-in-oil-in-water (W1/O/W2) emulsion method [50,51]. However, this method generally leads to small particle sizes, wide size distribution, *etc.* Dripping as another method [52,53] involves the formation of droplets from a water-in-oil (W1/O) emulsion by extrusion through a nozzle. The droplets are then solidified by subsequent processing. Although dripping method generally leads to uniform particle sizes, the formed microspheres tend to be quite large (up to 2 mm). In order to produce microspheres with reduced particle sizes ranging from 5 μm to 1,000 μm, ultrasonic excitation (or called ultrasonic vibration) [54,55] and electrostatic field pulling (or called electrospraying, electrostatic extrusion, or electrostatic atomisation, *etc.*) [56,57] have been used jointly with the dripping method. A further improvement is the use of a three flow channel device, where the controlled delivery of the initial water (W1) phase into the oil phase is achieved; instead of random mixing to form the (W1/O) emulsion, the W1 phase is slowly dripped into isolated droplets, which are then carried by the oil (O) phase [58]. The (W1) containing O phase is then isolated into droplets and carried by a second water phase (w2), leading to final microspheres with a controllable hollow interior. Similarly, porous polymer microspheres can also be prepared by a T-junction microbubbling device [59]. However, in a co-axial electrohydrodynamic microbubbling method [60], where an electric field is applied, the formed hollow microspheres tend to be too fine to be used for scaffold assembly and the fine microspheres have poor homogeneity in particle size. Last but not least, a tri-needle coaxial electrohydrodynamic device has been used to produce small multilayered polymeric microspheres loaded with a model protein—bovine serum albumin (BSA) [61]. Indeed, microspheres (especially porous ones) can be loaded with drugs, growth factors, and/or living cells before being assembled into functionally graded porous scaffolds.

## 6. Closing Remarks

The concept of graded/gradient materials has led to the development of graded/gradient porous biomaterials. While there are not many examples of actual applications of the graded/gradient porous biomaterials/implants, the interest of searching a new processing method of the materials still continues. So far, many fabrication methods have been developed for producing graded/gradient porous biomaterials. All biomaterials can be made into graded/gradient porous. The structural features and mechanical properties of graded/gradient porous biomaterials have been characterised. There are also some *in vitro* and *in vitro* studies of graded/gradient porous biomaterials. However, graded/gradient porous biomaterials are largely still in the stage of laboratory research. While there are several patents on graded/gradient porous biomaterials, examples of clinic applications are rare. The hurdle may be the manufacturing cost, which will prevent the realisation of the potential of graded/gradient porous biomaterials. For bioinert porous biomaterials, graded/gradient pore size and porosity will have obvious long term benefit in terms of tissue ingrowth and minimisation of stress shielding. However, for biodegradable porous biomaterials, the effect of graded/gradient porous structures on the tissue ingrowth and regeneration would be only in a short term.

While graded/gradient porous biomaterials and their composites have optimised mechanical and cell/tissue ingrowth properties, there is still room for improvement. Cost-effective methods are still needed to ensure suitable pore sizes and a high pore interconnectivity. With the new trend in tissue regeneration rather than tissue replacement, bioactive/biodegradable polymer/ceramic composites in the form of graded/gradient porous structures will have their advantages compared to homogeneous porous biomaterials and compared to sole polymer or ceramic porous materials. The pores in the graded/gradient porous parts can be further modified with coatings by means of slurry dipping, sol-gel coating, or biomimetic coating from the simulated body fluid. The pore surfaces can also be modified by grafting with proteins or embedding with growth factors. The pores can also be modified by filling or loading with biodegradable ceramics such as calcium phosphate cements, biodegradable polymers, or natural species like collagens, chitosan, cells, *etc.* One promising case would be the development of porous bioactive/biodegradable composites with graded/gradient pore size, porosity, and composition and the modification of the composites with spatially distributed biochemical stimuli so that stem cells loaded into scaffolds would develop into complex tissues such as bone-cartilage tissue.
